# Psychometric evaluation of a lifetime version of the marijuana problems scale

**DOI:** 10.1016/j.abrep.2018.05.001

**Published:** 2018-05-18

**Authors:** David C. Hodgins, Jonathan N. Stea

**Affiliations:** University of Calgary, Canada

**Keywords:** CIDI, Composite International Diagnostic Interview, CUD, cannabis use disorder, DSM, diagnostic and statistical manual of mental disorders, IDD, Inventory to Diagnose Depression, MMM, Marijuana Motives Measure, MPS, Marijuana Problems Scale, MPS-L, Marijuana Problems Scale - Lifetime, Marijuana problems scale, Cannabis problems, Marijuana, Psychometrics

## Abstract

**Introduction:**

The Marijuana Problems Scale (MPS) is a widely-used self-report measure of cannabis-related negative consequences that has a past three-month reporting window. This report describes the psychometric characteristics of a lifetime version (MPS-L).

**Methods:**

As part of a larger study, 119 individuals who had recovered from cannabis use disorder completed the MPS-L on two occasions 2 weeks apart and 91 participant-nominated family and friends also completed a collateral version of the scale.

**Results:**

Item analyses and principal component analysis (PCA) revealed that three of the 19 items were relatively weaker. Omitting these items, the MPS-L showed good internal reliability (α = 0.88, for summed severity total, α = 0.85 for number of consequences identified) and test-retest reliability (*r* = 0.81 and 0.73). As expected, correlations with collateral reports were moderate (*r* = 0.33 and 0.29), and collaterals reported significantly fewer negative consequences than participants. MPS total scores also correlated as expected with external validity measures (e.g., number of cannabis use disorder symptoms reported, motives for use, lifetime depression, treatment history). PCA supported the use of a total score summed score, but also revealed two secondary factors, measuring internal consequences (e.g., low self-esteem) and external consequences (e.g., financial difficulties).

**Conclusions:**

These analyses provide good preliminary support for a lifetime version of the MPS, with the summed severity total score performing slightly better than the total number of consequences endorsed.

## Highlights

• The psychometric qualities of a lifetime version of the Marijuana Problems Scale (MPS-L) were assessed.
• Two scoring methods for the MPS-L total score showed good internal and two-week test-retest reliability.
• The summed severity total score performing slightly better than the total number of consequences endorsed.
• Collateral reports from family and friends and responses to a variety of related measures supported the validity of the MPS-L.

## 1 Introduction

Cannabis is the most frequently used illicit substance in the world (Hall, Renstrom, & Poznyak, 2016), although some jurisdictions have begun to legalize and regulate its use (e.g., Canada, California, Colorado). Although most users do not experience extensive negative consequences, harms do occur for some individuals. Moreover, 0.5% of the adult population worldwide meet diagnostic criteria for cannabis use disorder (CUD), with rates in Canada, the USA, and Australia ranging from 1 to 2% (Hall et al., 2016).

A number of instruments have been developed to assess a variety of constructs related to CUD, including use, motives for use, problem severity, and diagnostic criteria for CUD (Rohsenow, 2008). The Marijuana Problems Scale (MPS) is a widely used instrument that assesses negative consequences of cannabis use (Stephens, Roffman, & Curtin, 2000). The MPS has a reporting window of the past three months, which makes it a useful index of current problems. However, a parallel lifetime version of the scale also has potential utility in supplementing measures of lifetime severity of cannabis use disorder, to assess level of lifetime problems relative to current problems within samples, and to facilitate comparisons of the severity level of samples recruited for different studies. This report provides some preliminary psychometric description of a lifetime version of the MPS.

The MPS was developed by Stephens and colleagues for their research program on cannabis treatment by adapting items from other drug use severity instruments (Stephens et al., 2000; Stephens, Roffman, & Simpson, 1994). The 19 items, representing various negative consequences, are rated as no problem (0), minor problem (1), or serious problem (2) over the past three months. The scale can be scored as a total count of problems (number of items marked as minor or serious, 0–19) or as a summed total of the severity ratings (0–38). Psychometric evaluation of the MPS has focused mostly on internal reliability, which has been demonstrated to be high in treatment and community samples (Hayaki, Anderson, & Stein, 2016; Stein, Caviness, & Anderson, 2013; Stephens et al., 2000; Stephens et al., 2004). Importantly, the MPS can be used to assess change over time. The number of problems score has been shown to be responsive to change as a treatment outcome measure, showing significant reductions post-intervention (Stephens et al., 2000).

The three-month version of the MPS continues to be widely used in cannabis research, despite limited further psychometric evaluation (Rohsenow, 2008). To date, no lifetime version of the MPS has been developed that can be used to assess lifetime problem severity. The current report provides a psychometric examination of a lifetime version of the MPS (MPS-L) that was administered as part of a descriptive study of recovery from CUD (Stea, Yakovenko, & Hodgins, 2015). Preliminary analyses examined the adequacy of the items using item-total correlations and examination of principal component loadings. To assess reliability of the revised scale, internal reliability was assessed and a re-test assessment of participants was conducted two weeks later. To assess convergent validity, a collateral sample of friends and family completed MPS-L describing the participant concurrently and comparisons were made with scales assessing other dimensions of cannabis problems. Specifically, we predicted a moderately size relationship between the MPS-L total scores and the number of DSM-5 CUD symptoms (*r* = 0.70 or greater), and a lower but significant relationship with the collateral report on the same items (*r* = 0.30–0.40). Given the comorbidity between depression and cannabis use disorders (e.g., Stinson, June Ruan, Pickering, & Grant, 2006), we predicted a moderate relationship with lifetime depression severity (r = 0.30–0.40). We also predicted that individuals who reported having received treatment for their cannabis problems would score significantly higher on the MPS-L than individuals who recovered without using treatment because treatment seeking is associated with greater problem severity (Stea et al., 2015). Finally, in terms of predictive validity, we expected that various motives for using cannabis scores would be differentially associated with the MPS- L, with use to cope predicted to have the strongest relationship, based upon previous research (Benschop et al., 2015). Finally, factor structure of the item pool was re-visited.

## 2 Method

### 2.1 Participants and procedure

Media recruitment in a mid-sized Canadian city was used to obtain a sample of 119 participants who had recovered from a CUD (American Psychiatric Association, 2013). The sample was 70% male and 30% female with a mean age of 37.3 years (SD = 12.9). The sample was predominately Caucasian (80%; Aboriginal 5%, other 15%). In terms of employment, 52% were employed full-time, 10% part-time, 20% were students, and 11% were unemployed. The mean age of onset of CUD was 20.0 years (SD = 6.5) and the median length of recovery was 5 years. Sixty-eight (57%) were currently abstaining from cannabis use (i.e., had not used cannabis for the past 12 months), and the remainder reported some non-problematic use (for more detail see (Stea et al., 2015)). Formal treatment involvement was reported by 53 participants (45%).

Participants were interviewed in person and were also asked to nominate a family member or friend to act as corroborator, who was interviewed by telephone by a research assistant who was blinded to the participant's initial assessment information. Collaterals were contacted for 91 participants (77%) and interviews were conducted a mean of 10.3 days after the participant interview (SD = 7.7). The collaterals included 21 partners, 17 parents, 17 friends, 3 children, and 6 other types of family members. For the remaining 28 participants, 7 did not provide a collateral, 14 provided names but the collateral was unable to be contacted, and 7 provided names that were contacted but were unwilling to participate. Collaterals provided responses to the MPS-L items and were also asked to rate their confidence in responding to the MPS-L items overall (1 = very uncertain, 2 = uncertain, 3 = certain, or 4 = very certain). The mean confidence score was 3.2 (SD = 0.9), with a mode of 4 and a range of 1 to 4.

Participants were re-contacted within two weeks after their initial interview to assess test-retest reliability of their self-reported cannabis use as well as responses to a variety of the assessment instruments (Stea, 2013). Telephone interviews were conducted by research assistants who were blinded to the participants' initial assessment. The mean number of days for the 107 participants successfully re-interviewed was 9.6 (SD = 13.1).

Ethical approval was provided by the Conjoint Faculties Ethics Review Board at the University of Calgary.

### 2.2 Measures

The MPS was modified to inquire whether each item had ever been experienced as a problem, using the same response options as the three-month version (0 = no, 1 = minor, 2 = serious). Two summary scores were calculated, a total summed score of the severity ratings (MPS-severity, 0–38) and a total problem score (MPS-number, the number of items scored minor or serious, 0–19).

Lifetime cannabis use disorder was assessed using the Composite International Diagnostic Interview (CIDI) (Kessler & Ustun, 2004), which we updated to assess DSM-5 symptoms and criteria (American Psychiatric Association, 2013). The CIDI is a widely used structured interview designed to be administered by trained laypersons. It has been validated against the Structured Clinical Interview for the DSM-IV (First, Spitzer, Gibbon, & Williams, 2002). The categorical CUD diagnosis was used to assess participant eligibility for the study, and the lifetime symptom count (0−11) was used as an external validity measure.

There were three additional external validity measures used in this report. The Marijuana Motives Measure (MMM) (Simons, Correia, Carey, & Borsari, 1998) provides five motive subscales with respect to lifetime cannabis use: Enhancement, coping, social, conformity, and expansion. Each subscale is measured with five items, and the scale reliability and validity has been replicated with adult samples (Benschop et al., 2015).

Lifetime depressive symptoms were measured with the lifetime version of the 19-item Inventory to Diagnose Depression (IDD) (Zimmerman, 1994). The participant is asked to describe the worst week of their lives when they felt most depressed. The lifetime version total score has good test-retest reliability and discriminative validity (Sakado, Sato, Uehara, Sato, & Kameda, 1996; Sato et al., 1996).

Treatment involvement was assessed by asking whether the participant had ever received at least one session of formal or professional treatment for a cannabis use problem (e.g., talking to a physician, counsellor or therapist, calling a helpline, or receiving medication). The interviewer probed and clarified responses. Involvement in self-help groups was excluded.

### 2.3 Analyses

As a preliminary step, corrected item-severity total summed scores correlations and principal component analysis of the item pool were computed to identify any weak items. Next, descriptive analyses of each of the MPS-L items and total scores were conducted. To assess internal reliability, coefficient alpha was conducted for both the summed severity total and the number of symptoms total. Internal reliability scores of 0.80 and greater are considered good. For test-retest reliability, Pearson correlations were computed for each MPS-L item and the total scores. To assess the validity of the total scores, Pearson correlations were computed with the number of lifetime DSM-5 symptoms (CIDI), the five MMM scales, and the lifetime severity of depression score (IDD). *T*-tests were used to compare the MPS-L total scores for participants who had or had not attended cannabis treatment. Pearson correlations were conducted between participants and collateral reports for each MPS-L item and total scores.

Finally, a principal component analysis was conducted to explore the factor structure of the scale. The ordinal responses to the items were used in the polychoric correlation matrix. Two methods were used to determine the number of components to rotate: Cattell's scree test, and Horn's parallel analysis (O'Connor, 2000). Varimax rotation was used to examine simple structure.

## 3 Results

Initial examination of corrected item-summed severity total score correlations (Table 1)) revealed three weak items, with correlations of 0.3 or lower: medical problems, legal problems and blackouts. These same items showed the lowest loadings on the first unrotated factor in an initial principle components analysis (not shown). These items were omitted from further analyses and from the MPS-L.

**Table 1:** Initial endorsement and test-retest reliability of individual items.

Item	Endorsement (%)			Item-total r	Test-retest r
	No	Minor	Serious		
Problems between you and your partner	34	25	40	0.37	0.69
Problems in your family	29	33	38	0.52	0.71
Neglect in your family	31	28	41	0.69	0.67
Problems between you and your friends	46	32	22	0.57	0.56
Missed appointment, work, or, class	35	26	39	0.56	0.59
To lose a job	72	13	15	0.44	0.54
To have lower productivity	24	40	36	0.53	0.53
Medical problems *	69	15	16	0.25	0.64
Withdrawal Problems	34	39	28	0.53	0.76
Blackouts or flashbacks *	75	16	9	0.30	0.57
Memory loss	25	45	29	0.39	0.64
Difficulty sleeping	60	24	16	0.36	0.68
Financial difficulties	50	23	26	0.53	0.74
Legal problems *	72	12	16	0.19	0.65
Lower energy level	19	38	43	0.51	0.69
Feel bad about use	30	32	38	0.63	0.60
Lower self-esteem	37	22	41	0.61	0.64
Procrastinate	12	24	65	0.60	0.64
Lack of self-confidence	40	19	40	0.56	0.56

Note: * item excluded from scale.

The internal reliability for the remaining 16 items, assessed with Coefficient alpha, for both total scores was good for both total scores (MPS-severity, 0.88; MPS-number, 0.85). The total scores correlated highly, *r* = 0.94. Table 1 displays the proportion of participants endorsing each response option for each item of the MPS-L as well as the test-retest correlations for individual items. Test-retest correlations ranged from 0.53 to 0.76 for individual items. Both total scores showed good test-retest reliability, with the summed severity score being slightly higher than the number of problems score (*r* = 0.81 vs. 0.73). The total scores did not differ significantly from the initial to retest administration (MPS-severity, M = 15.9 (7.6) and 15.0 (8.6), *t*(104) = 1.96, *p* < .06; MPS-number M = 10.4 (4.0) and 9.8 (4.4), *t*(106) = 1.89, *p* < .07).

Table 2 displays the means and standard deviations for the external validity measures and their correlations with the MPS-severity and MPS-number scores. As expected, both MPS-L scores were highly correlated with the number of CUD symptoms, and moderately correlated with lifetime depression severity. The relationship with all motives was significant but, as expected, the MPS-L -coping relationship was the strongest. Participants with a history of treatment versus those who recovered without treatment scored higher on both the MPS-severity (M = 18.8 (7.2) vs. 13.3 (7.4); *t*(117) = 4.07, *p* < .0001)) and the MPS- number (M = 11.6 (3.9) vs. 9.1 (3.9); *t*(117) = 3.53, *p* < .001)).

**Table 2:** Means (standard deviations) of validity measures and Pearson correlations with MPS-L total scores.

Measure	M	SD	r_severity_	r_number_
CIDI Lifetime cannabis use disorder symptoms	8.6	2.6	0.67	0.69
MMM social	17.4	5.2	0.24	0.25
MMM coping	16.5	5.7	0.54	0.50
MMM enhancement	20.2	3.9	0.19	0.20
MMM conformity	11.1	5.6	0.42	0.38
MMM expansion	14.2	5.8	0.26	0.28
IDD lifetime depression severity	43.8	16.5	0.40	0.31
Collateral MPS-L_severity_	11.8	8.3	0.33	–
Collateral MPS-L_number_	8.2	4.6	–	0.29

Note. MPS-L = Marijuana Problem Scale- Lifetime; MMM = Marijuana Motives Measure; IDD = Inventory to Diagnose Depression. All correlations significant at *p* < .05 except where indicated as non-significant (ns).

Table 2 also displays the mean collateral MPS-L total scores. The collateral MPS-L -severity and MPS-L-number scores were significantly lower than the same scores produced by the participants, *t*(90) = 3.85, p < .001 and *t*(90) = 3.3, p < .001, showing that collaterals were aware of fewer difficulties than participants. The Pearson correlations between collaterals and participants was *r* = 0.33, *p* < .001, for MPS-L-severity and *r* = 0.29*, p* < .007 for MPS-L-number.

The principal components analyses showed that the Kaiser-Meyer-Olkin Measure of Sampling Adequacy was good (0.83) and all items loaded on the first unrotated component (0.41 to 0.75, M = 0.52), which suggests that all items are measuring a similar construct. Both the Scree test and the Parallel test indicated rotation of two factors, which together accounted for 46% of the variance. The Varimax rotated loadings are displayed in Table 3. Examination of the loadings show that one factor measured less severe and more common effects (e.g., low self-confidence, lower energy) and the other more severe and less common effects (e.g., loss of job, financial difficulties). Two items, memory problems and neglect of family, loaded on both factors. Excluding these, the coefficient alpha for the summed severity totals for the two subscales was 0.85 (internal, 7 items) and 0.77 (external, 7 items). The two subscales were moderately correlated with each other (*r* = 0.59). Mean scores on factor one (M = 1.08, SD = 0.57) were significantly higher than scores on factor two (M = 0.73, SD = 0.47), which is consistent with the interpretation that these effects are more common, *t*(118) *=* 7.59, *p* < .0001).

**Table 3:** Rotated component loadings of MPS-L items.

Item	Component	
	1	2
To lack of self-confidence	**0.810**	0.091
Lowered self-esteem	**0.796**	0.145
To feel bad about your use	**0.739**	0.230
To procrastinate	**0.657**	0.334
To have lower energy level	**0.626**	0.225
To have lower productivity	**0.535**	0.360
To neglect your family	**0.534**	**0.524**
Difficulty sleeping	**0.470**	0.149
Memory loss	0.337	0.311
To lose a job	−0.006	**0.741**
Problems between you and your friends	0.160	**0.707**
To miss days at work or miss classes	0.282	**0.648**
Financial difficulties	0.272	**0.632**
Problems with your family	0.241	**0.589**
Withdrawal symptoms	0.312	**0.498**
Problems between you and your partner	0.166	**0.434**

Note. MPS-L = Marijuana Problem Scale- Lifetime. Loadings greater than 0.40 are bolded.

## 4 Discussion

The lifetime version of the MPS shows acceptable psychometric qualities in this community sample of individuals with a past CUD. The scale items measure common and less common negative consequences of cannabis use, but the total score is a reliable indicator of an overall level of problems. Although the scores for both the summed severity total and the total number of problems endorsed performed reasonably well and are highly correlated, the severity score generally was slightly stronger. This finding makes sense as the severity total captures the level of impact whereas the number of problems total score equates items having minor and major effects. Unless there is particular interest in the number of problems endorsed, it is advisable for researchers to use the MPS-L severity total score. Clarity in published reports about which scores are used in important. In previous research, the method of computing a total score for the three-month version of the MPS is sometimes not readily apparent.

Participants showed good test-retest reliability at the individual item level and, in particular, for the total scores. Collaterals tended to report fewer negative consequences than participants (e.g., about two fewer problems on MPS-L number total) and the total scores were only moderately correlated. This finding is not unexpected as collaterals tend to be less aware of some negative effects, and most of the negative consequences would have occurred a number of years prior given that all participants were in recovery from CUD.

Preliminary analyses revealed that three of the 19 original items seemed relatively weak, and were therefore excluded in the MPS-L. One of these items concerns blackouts, which is not a typical effect of heavy cannabis use. The MPS items have also been used to measure negatives effects of alcohol, so it is likely that this item was useful in that context. The other weak items were medical and legal problems, which both have some face validity for cannabis. The term “medical problems” is vague and may also be more associated with alcohol use (e.g., liver, withdrawal seizures, etc.). Legal problems are culturally influenced and vary from jurisdiction to jurisdiction, and over time. Because of this, experiencing legal problems related to substance use was dropped as diagnostic criteria in the DSM-5 for all substance use disorders and gambling disorder.

Unfortunately, this investigation only included individuals who were no longer experiencing CUD, however, it is likely that the scale would perform as well, or even better with people reporting on more recent effects. Nonetheless, this should be investigated in future studies. It is also important to note that sample was comprised largely of middle-class Caucasian Canadians. Psychometric analyses of the scale in other populations is important. As well, this study did not examine content validity. Whether additional items should be included in a scale comprehensively measuring negative consequences could also be further investigated. Despite these limitations, these analyses provide preliminary support for a lifetime version of the MPS-L. An index of negative consequences of cannabis use with a lifetime reporting frame has not previously been available.

## Role of funding sources

This research did not receive any specific grant from funding agencies in the public, commercial, or not-for-profit sectors, although JS received scholarship support from the Canadian Institutes of Health Research, Alberta Innovates Health Solutions, and the Killam Trusts.
